# Fitness of *Isidorella newcombi* Following Multi-generational Cu Exposures: Mortality, Cellular Biomarkers and Life History Responses

**DOI:** 10.1007/s00244-022-00931-w

**Published:** 2022-04-20

**Authors:** R. P. Ubrihien, W. A. Maher, A. M. Taylor, M. M. Stevens, T. Ezaz

**Affiliations:** 1 Centre for Applied Water Science, Institute for Applied Ecology, University of Canberra, Canberra, ACT 2601 Australia; 2 Research School of Earth Sciences, Australian National University, Canberra, ACT 2601 Australia; 3 NSW Department of Primary Industries, Yanco Agricultural Institute, Private Mail Bag, Yanco, NSW 2703 Australia; 4 Graham Centre for Agricultural Innovation NSW Department of Primary Industries, Charles Sturt University, Wagga Wagga, Australia; 5 Centre for Conservation Ecology and Genomics, Institute for Applied Ecology, University of Canberra, Canberra, ACT 2601 Australia

## Abstract

The effects of multigenerational Cu exposure on the freshwater gastropod *Isidorella newcombi* were investigated*.* Snails were exposed to a range of treatment-specific Cu concentrations in the parental to F_2_ generations, and a common Cu concentration in the F_3_ generation. In the parental to F_2_ generations, some general responses to 3 days Cu exposures included reduced survival and feeding in snails exposed to higher Cu concentrations. This suggested that the snails exposed to the high Cu concentration were experiencing Cu-induced stress that may apply selection pressure. In the F_3_ generation, when all treatments were exposed to a common Cu concentration, increased survival was correlated with the pre-exposure Cu concentration history. Snails that had been pre-exposed to Cu also displayed reduced stress at a sub-lethal level, indicated by lower lysosomal destabilisation (LD). Mortality and LD responses in the F_3_ generation were not related to Cu tissue concentrations, indicating increased tolerance and reduced stress were not related to changes in Cu bioaccumulation. Total antioxidant capacity increased in the higher Cu concentration pre-exposure treatments which could be associated with lower Cu-induced stress, however, this is not supported by the oxidative damage marker lipid peroxidation, which also increased. While Cu tissue concentrations and oxidative stress markers were assessed to determine underlying reasons for increased tolerance in snails from a population with a multi-generational exposure history to Cu, the results were not conclusive. Despite this, it was demonstrated through increased survival and reduced LD that Cu tolerance can develop over a short evolutionary time scale.

**Supplementary Information:**

The online version contains supplementary material available at 10.1007/s00244-022-00931-w.

The concentration of copper (Cu) is elevated in the biosphere because of mining, processing and transport, as well as the widespread industrial, domestic and agricultural use of Cu (Wright and Welbourn 2002). In Australia, there are reported instances of Cu contamination associated with mining activity and with the use of Cu as an agricultural pesticide over extended periods (Merry et al. 1986; Klessa et al. 1997; Eriksen et al. 2001; Stevens 2002). The persistence of Cu in the environment can cause high Cu concentrations to accumulate in areas where it is extracted, processed or used (Kakkar and Jaffery 2005). The widespread use of Cu and its persistence in the environment provide the potential for plant and animal populations to be exposed to elevated concentrations over multiple generations.

A population’s phenotypic level of resistance to a contaminant will be determined by its genetics and multi-generational exposure history (Morgan et al. 2007). When populations are exposed to contaminants that exert selection pressures over multiple generations, rapid evolutionary change can occur (Hoffmann and Hercus 2000). The microevolution which can occur as a result of multi-generational exposure can result in populations with increased tolerance to the contaminant (Medina et al. 2007). While adaptations associated with multi-generational contaminant exposure can result in increased tolerance in contaminated environments, biological trade-offs in resistant populations in the form of reduced fitness in uncontaminated environments have also been reported (Monaghan et al. 2009; Mireji et al. 2010).

The classic dose–response framework often used in risk assessment relies on the assumption of a consistent underlying response that assumes genetic uniformity across populations. Populations previously exposed to contaminants, however, often show a different dose–response to unexposed populations (Coutellec and Barata 2011). To account for adaptation in risk assessment, there is a need to quantify the adaptive potential of populations (Côte et al. 2015). This is particularly true for contaminants such as Cu that are persistent in the environment and have the potential to exert toxic effects over multiple generations.

While experiments have been conducted to investigate the development of resistance to Cu in aquatic organisms, these studies have used exposure to consistent concentrations over entire lifecycles (Peña and Pocsidio 2007; Rogevich et al. 2008). In aquatic environments, excess bioavailable Cu resulting from contamination events is often only present for a short period due to chemical complexation, precipitation and adsorption (Flemming and Trevors 1989). As a result, the exposure of organisms in some Cu-contaminated environments may be in short pulses that are associated with specific contamination events. An understanding of the effects of Cu over multiple generations of short exposures would increase the understanding of the multigenerational effects of Cu on organisms exposed to environmentally realistic scenarios.

The Australian endemic freshwater gastropod *Isidorella newcombi* has been identified as a pest in Australian rice growing areas and is controlled by application of CuSO_4_ (Stevens et al. 2014). Under this scenario, there is the potential for multi-generational exposure of this snail to elevated Cu concentrations and a resultant increased Cu tolerance. Freshwater snails form important links within freshwater ecosystems by transferring energy and materials through food webs (Habdija et al. 1995; Lagadic et al. 2007). Freshwater snails also have desirable characteristics for investigation of the multigenerational effects of contaminants, as they are easy to culture, reach reproductive age early, reproduce continuously and have a short embryonic development period (Ravera 1977). The association with Cu in rice growing areas, as well as its desirable characteristics for investigating multi-generational exposures, makes *Isidorella newcombi* an ideal model species for the investigation of the effects of multigenerational Cu exposures.

*Isidorella newcombi* were exposed to Cu over four generations. The aim of the study was to investigate if there is any development of resistance and, if so, if there were any associated biological trade-offs over these generations. The effects of Cu on survival, fecundity, embryo development and juvenile survival were assessed across multiple exposure concentrations in the parental, F_1_ and F_2_ generations. In the F_3_ generation, all treatments were exposed to a common Cu concentration, and the same responses were compared with the specific objective of determining the extent to which differing pre-exposure histories led to increased tolerance to Cu. The bioaccumulation of Cu and the oxidative stress responses were also investigated in the F_3_ generation to determine if the biological mechanisms associated with differential Cu tolerances across treatments could be identified. Biological trade-offs were also investigated through measuring fecundity of the F_3_ generation and the viability of the F4 eggs and juvenile snails.

## Methods

### Snail Cultures and Experimental Organisms

Snails were sourced from an in-house culture maintained at a water temperature of 22 ± 1 °C and a 12/12 day-night light cycle. The original *I. newcombi* were sourced from uncontaminated wild populations at Yanco, New South Wales (34^o^37′28″ S 146^o^25′05″ E). The snails were maintained in natural water sourced from the Cotter River at Vanities Crossing, ACT (35° 20′ 37″ S 149° 4′ 59″ E; Cu 2.4 µg L^−1^, pH 6.7, conductivity 0.044 mS cm^−1^, turbidity 1.4 NTU, hardness 6.6 mg L^−1^, salinity 0.02 ppt and TOC 1.46 mg L^−1^). At all stages during the study, aerators were used to maintain oxygen levels in the experimental solutions at ≈ 100% oxygen saturation. The only exception to this was in the small containers used for individual egg masses and juveniles to 6 days old, as they were too small to aerate without causing excessive turbulence. The snails were fed lettuce leaves washed in the same river water in which they were maintained.

### Exposure Conditions and Experimental Design

**Fig. 1:**
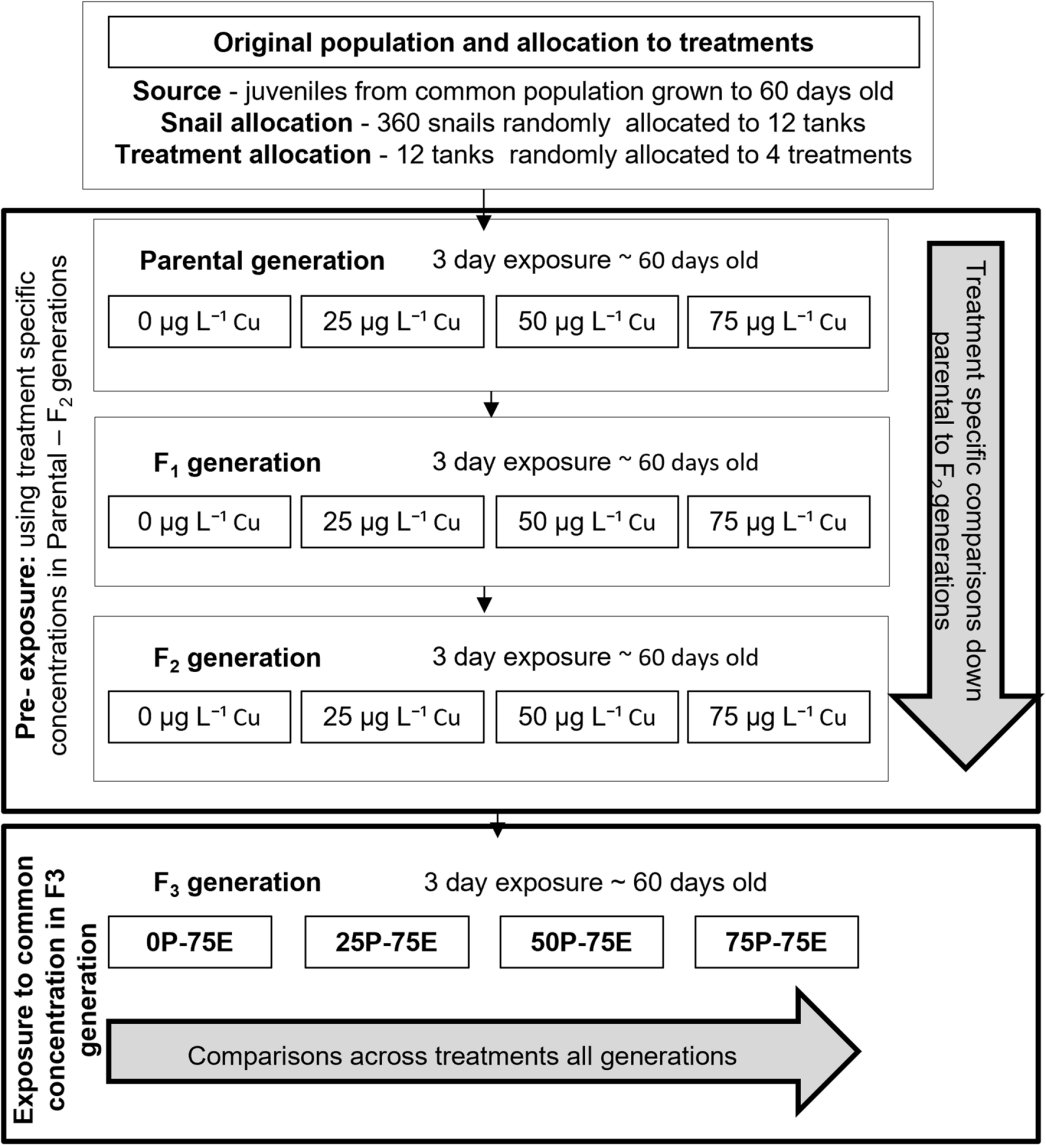
Experimental design for the multigenerational exposure of *Isidorella newcombi* to Cu. Treatment specific concentrations are included in the boxes within the figure for the parental, F_1_ and F_2_ generations. In the F_3_ generation all treatments were exposed to 75 µg L^−1^ Cu. In the F_3_ generation, P = pre-exposure (µg L^−1^ Cu treatment the snails were exposed to in the parental, F_1_ and F_2_ generations), and E = exposure, which is the concentration in µg L^−1^ Cu that the snails were exposed to in the F_3_ generation. As the 100 µg L^−1^ Cu treatment did not continue through the experiment it is not included in the figure

Prior to the experiment, adult snails were kept in a large plastic aquarium for one week. All snails were then removed from the aquarium. The aquarium was refilled with water and clutches of eggs attached to the side of the tank were allowed to hatch. Once the juveniles were large enough to be handled, they were collected and divided into multiple three litre aquariums (PLA-House 1013, Oscar Enterprises, Gardena, CA, USA) at equal densities. After 56 days, 450 individuals were selected and separated into 15 three litre plastic aquariums (PLA-House 1013) each containing 2 L of river water and 30 snails. Each aquarium was randomly allocated to one of five treatment groups with three replicate aquariums per treatment. A total of 90 snails were used per treatment per generation. After separation into replicate-specific aquariums, the snails were acclimated to the aquariums under control conditions for 1 week prior to Cu exposure. Snails were exposed to Cu just prior to reproductive maturity, as juvenile life stages are typically the most sensitive, and for the purposes of this study, the snails were exposed to relatively high Cu concentrations of up to 100 µg L−^1^. In addition, exposing snails prior to reproductive maturity ensured that all subsequent offspring were from snails that survived the treatments. Prior to the experiment, the minimum time to first reproduction observed in other cultures maintained under experimental conditions was approximately nine weeks. On this basis, exposure of the snails to Cu commenced at 60 days. The experimental design is shown in Fig. 1. The snails were exposed to treatment Cu concentrations for 3 days, while the control snails were kept in clean Cotter River water. The treatment groups had nominal concentrations of 25, 50, 75 and 100 µg L^−1^ Cu added (CuSO_4_.5H_2_O (Univar® Downers Grove)). Three replicate containers with 30 snails were used for each of the treatment levels and the control. Fifteen grams of lettuce (wet weight) was added to each replicate at the start of the three-day exposure period. During the exposure period, all aquariums were checked regularly, and dead snails were removed to minimise the chance of fouling. Due to high mortality in the 100 µg L^−1^ Cu treatment, this treatment was discontinued after the parental generation and is not discussed further. The measured Cu concentrations in the control, 25, 50 and 75 µg L^−1^ Cu treatments were 4 ± 1, 28 ± 1, 53 ± 2 and 80 ± 1 µg L^−1^ Cu, respectively. The Cu concentrations were measured at the start of the exposure period and no water changes were made during the 3 days exposure. After the exposure period, the snails were transferred to a clean aquarium containing Cotter River water for 14 days to allow them to recover prior to fecundity and juvenile survival assessment. The exposures of the F_1_ and F_2_ generations were conducted in the same manner, with the juvenile snails from each treatment grown to 60 days of age and again exposed to treatment-specific Cu concentrations. In the F_3_ generation, the same design was used, with the exception of the Cu exposure concentrations. All treatments were exposed to a nominal concentration of 75 µg L^−1^ Cu in the F_3_ generation. In the F_3_ generation, the naming of treatments follows a protocol of *x*P-75E, where *x* refers to the exposure concentrations for the treatment in the parental, F_1_ and F_2_ generations, 75E is the exposure concentration in the F_3_ generation. An F_4_ generation was produced to allow an assessment of hatching rates and juvenile survival.

### Food Consumption

As the wet mass of lettuce increases when put in water for 72 h, an average mass for 15 g initial mass of lettuce left in water for 72 h was determined. Four aquarium tanks were set up identical to the exposure aquariums, but no snails were added. The post-immersion mass of the lettuce from these four aquariums was used as a starting point to calculate lettuce consumed by the snails. The lettuce remaining at the end of the exposure period was carefully removed by hand and weighed. Lettuce consumed in each replicate was calculated by subtracting the lettuce remaining in the replicate at the end of the exposure from the average post-immersion mass of the lettuce from these four aquariums. There was some variability in the data, and if the calculated value for a replicate was negative, zero lettuce consumption was assumed. The lettuce consumption was normalised to lettuce consumption per snail per day to adjust for mortality during the exposure period.

### Fecundity

After each exposure, surviving snails were given a 14 days recovery period, prior to being transferred to 770 mL polypropylene containers (Chanrol C30). The snails from each treatment replicate were divided between two containers to reduce their density. The containers were inspected daily, and the egg clutches were marked and any adult mortality recorded. Every 3 days snails were transferred to new containers, the number of eggs in each clutch was counted, and the clutches were transferred to individual polypropylene containers (Chanrol 01PC1). All treatments were maintained under these conditions for 12 days (day 15 to day 26 following exposure) for the purposes of recording fecundity data and harvesting clutches for the assessment of offspring viability. While some treatments were maintained under these conditions for a longer period to ensure there were sufficient numbers for the next generation, only data from egg masses laid in the first 12 days were included in fecundity and offspring viability calculations.

### Hatching Success and Juvenile Survival

Once in their individual containers, the clutches were monitored every day until the day of first egg hatch, the number of hatched individuals from each clutch was recorded, and a small piece of lettuce (5 to 10 mm^2^) was added. Hatching success was calculated as the number of hatched snails divided by the total number of eggs in the clutch. Six days after hatching, the number of juvenile snails surviving in each container was counted. Juvenile survival to 6 days was calculated as the number of snails alive in the containers at 6 days divided by the total number of hatched snails. After they were counted at 6 days of age, all juvenile snails were then transferred to a treatment replicate-specific tank for approximately 54 days prior to the next Cu exposure.

### Exposures for Biomarkers

In the F_1_, F_2_ and F_3_ generations, a second set of exposures was completed for biomarker analysis. These snails were exposed to the same concentrations and conditions as the experimental treatments described above, the only difference being that the snails were exposed at ~ 100 days of age rather than at 60 days. The increase in age of the snails meant that adult snails were being exposed, rather than juveniles. This could not be avoided as the workload associated with the culture transfer and juvenile snail counts did not allow biomarker exposures to be done simultaneously.

### Metal Analysis

Copper concentrations were measured in the experimental media and the tissues of the F_3_ snails. Snails were depurated for 24 h in clean water, dissected, and the soft tissues removed from the shells. Soft tissues were lyophilised using a freeze dryer (Labconco, Freezone plus 6) and digested as per the procedure outlined in Baldwin et al. (1994). Total dry mass was recorded for all samples. If the samples had a dry mass greater than 0.07 g, the samples were homogenized and a subsample of 0.07 g was used for the digestion. The sample was placed into a 7 mL polytetra-flouroacetate (PFA) digestion vessel with 1 mL of nitric acid (67–70% Aristar BDH, VWR, USA). For every 20 samples, two certified reference materials (National Institute of Science and Technology 1566b) and two blanks were prepared, digested and analysed. The samples were digested at 600 W for 2 min, 0 W for 2 min and 450 W for 45 min in a microwave oven (MDS-2000, CEM Corporation, USA). Samples were then diluted to 1% v/v concentration of acid/tissue to deionised water using an auto-dilutor (Gilson GX 271, USA) for inductively coupled plasma-mass spectrometer (ICP-MS) analysis. Water used for dilution contained an ICP-MS mixed 7-element internal standard (EM Science) to monitor for variations due to instrument drift or matrix effects.

Diluted samples were analysed using a Elan DRC-e ICP-MS (Perkin-Elmer, USA) following the protocol described by Maher et al. (2001). Certified reference materials (National Institute of Standards and Technology Oyster Tissue 1566b) and blanks were analysed with samples to determine the recovery of metals. The certified value for NIST1566b is 71.6 ± 1.6 µg g^−1^ Cu dry mass and the measured value was 68.3 ± 0.5 µg g^−1^ Cu dry mass (*n* = 6) was and within the acceptable range. External calibration standards used for quantitation were made up from a 10 mg L^−1^ Reference Standard, ICP-MS Calibration Multi Element Standard 2 (AccuTrace, AccuStandard, USA) in 1% (*v*/*v*) HNO_3_ as 1, 0.1, 0.01 and 0.001 mg L^−1^ solutions. Recalibration was performed every 15 samples during analysis.

### Total Antioxidant Capacity, Lipid Peroxidation and Lysosomal Membrane Destabilisation

In the F_3_ generation, total antioxidant capacity (TAOC), lipid peroxidation (LP) and lysosomal membrane destabilisation (LD) were assessed. Nine snails from each treatment were used for each analysis, with three snails coming from each of the treatment replicates. Tissue preparations for TAOC and LP measured as thiobarbituric acid reactive substances (TBARS) analysis were prepared at the end of the exposure period. Snail tissues were removed from their shells. The digestive tract of the organism was then separated from the remaining tissue. The digestive tract tissue was homogenized in 500 µl of a 5 mM potassium phosphate buffer containing 0.9% sodium chloride and 0.1% glucose, pH 7.4 (1:5 *w*/*v*). The tissue was homogenized on ice using a motorized microcentrifuge pellet pestle, sonicated on ice for 15 s at 40 V and centrifuged (5804R centrifuge, Eppendorf, Austria) at 10,000 × *g* for 15 min at 4 °C. A 50 µl aliquot of supernatant was removed for TAOC and a 250 µl aliquot of supernatant was removed for TBARS, with the pellet and remaining supernatant being reserved for protein analysis. All three samples were stored at − 80 °C until analysed.

The TAOC of tissue lysates was measured using a Cayman chemical assay (Cayman Chemicals, Michigan, USA, #709,001). This assay is based on the ability of the antioxidants in the sample to inhibit the oxidation of 2, 2′-azino-di-[3-ethylbenzthiazoline sulphonate] (ABTS) to ABTS^**·**+^ by metmyoglobin. The assay was conducted as per the instructions outlined in the assay kit and absorbance read at 750 nm on a BioRad Benchmark Plus® microplate spectrophotometer. Lipid peroxidation (LP) was determined by measuring the thiobarbituric reactive substances (TBARS) present in the tissue lysates. The Oxitek® TBARS assay (Zeptometrix Corporation, Massachusetts, USA, #0,801,192) used is based on specificity of malondialdehyde (MDA), which is a by-product of lipid peroxidation. The assay was conducted as described in the assay kit and absorbances were read at 532 nm on a microplate spectrophotometer (BioRad, Benchmark Plus®). Detailed descriptions of the TAOC and LP assays can be found in the supplementary information (SI1).

Protein in samples was measured to provide a baseline for the normalisation of TAOC and MDA. Protein was quantified using the Fluoroprofile® Protein Quantification Kit (#FP0010; Sigma-Aldrich, USA), a fluorescent assay based on epicocconone. Fluorescence was read at 485 nm excitation and 620 nm emission wavelengths on a BioRad Benchmark Plus® microplate spectrophotometer. A bovine serum albumin (BSA) calibration curve was used to calculate protein concentrations.

The methods used for the lysosomal stability test were based on procedures developed by Ringwood et al. (2003). Each organism was dissected to isolate the digestive gland and all attached gonadal tissue was removed. The digestive gland was rinsed with calcium and magnesium free saline buffer (CMFS) (20 mM HEPES (Thermo-Trace C07133), 360 mM NaCl (UNIVAR F2A021), 12.5 mM KCl (M and B Laboratory Chemicals 20,116) and 5 mM tetrasodium EDTA (Merck 10,093.5 V)) pH 7.35–7.4. The digestive gland tissue was then homogenised with the use of a scalpel and glass Petri dish (inverted and filled with ice). Samples were then placed into a 24 well plate with 600 µL of CMFS on ice and shaken for 20 min at 100 rpm on an orbital shaker. A trypsin solution was made up using 1 mg of trypsin (Sigma 1426-1G) to 1 mL CMFS. 400 µL of the trypsin solution was then added to the sample and the sample was shaken for a further 20 min at 100 rpm on an orbital shaker. Samples were then sheared with a glass pipette and transferred to a microcentrifuge tube/filter apparatus. Samples were initially centrifuged at 200–225 × *g* (5 min, 15 °C). The filter was then removed, the supernatant discarded and the pellet resuspended in 1 mL CMFS. This solution was then centrifuged at 200–225 × *g* (5 min, 15 °C). If required, an additional resuspension and centrifugation step was used to remove debris from the cells. After the final rinse, the supernatant was discarded and the pellet was resuspended in 50–300 µL of CMFS dependent on the size of the pellet. A neutral red (Sigma-Aldrich, N-7005) stock solution was made up in dimethyl sulphoxide (Merck 10,323) at a concentration of 4 mg mL^−1^. A working solution was then prepared using the stock solution at a concentration of 10 µl mL^−1^ in CMFS. The working solution was added in 1:1 *v*/*v* ratio to the final addition of CMFS in the microcentrifuge tube, and the cells were incubated in the dark for 60 min. After the incubation period, a wet mount slide was prepared and observed at 400 × magnification under a light microscope. At least 50 cells were counted for each sample.

### Data Analysis

Initial data preparation was completed in Microsoft Excel 2010. All data analysis was completed using R 3.1.2 (R Core Team 2014). If assumptions of normality and homoscedasticity were met, single factor ANOVA with Tukey HSD post hoc analysis was used to compare treatments. If data did not meet these assumptions, a Kruskal–Wallis test was used with Dunn’s Test being used for multiple pairwise comparisons (Dinno 2016). Spearman’s rank order correlation was used to evaluate relationships between variables.

## Results

Within both the results and discussion sections, the parental, F_1_ and F_2_ generations will be presented separately to those for the F_3_ generation. This is because the parental to F_2_ generations used treatment-specific exposure concentrations in order to establish different pre-exposure histories across the treatments. Conversely, all treatments were exposed to a common concentration in the F_3_ generation as a means of testing the effect of the different pre-exposure histories on the response of the snails to Cu (see experimental design Fig. 1). Note that, while this section focusses on the parental to F_2_ generations, some analyses for the 75 µg L^−1^ Cu treatment include data from the F_3_ generation. As the exposure concentrations remained consistent from parental to F_3_ generation for the 75 µg L^−1^ Cu treatment, where appropriate for comparisons across generations within the 75 µg L^−1^ Cu treatment, the F_3_ data were included.

## Parental to F_2_ Generations

### Food Consumption

**Fig. 2:**
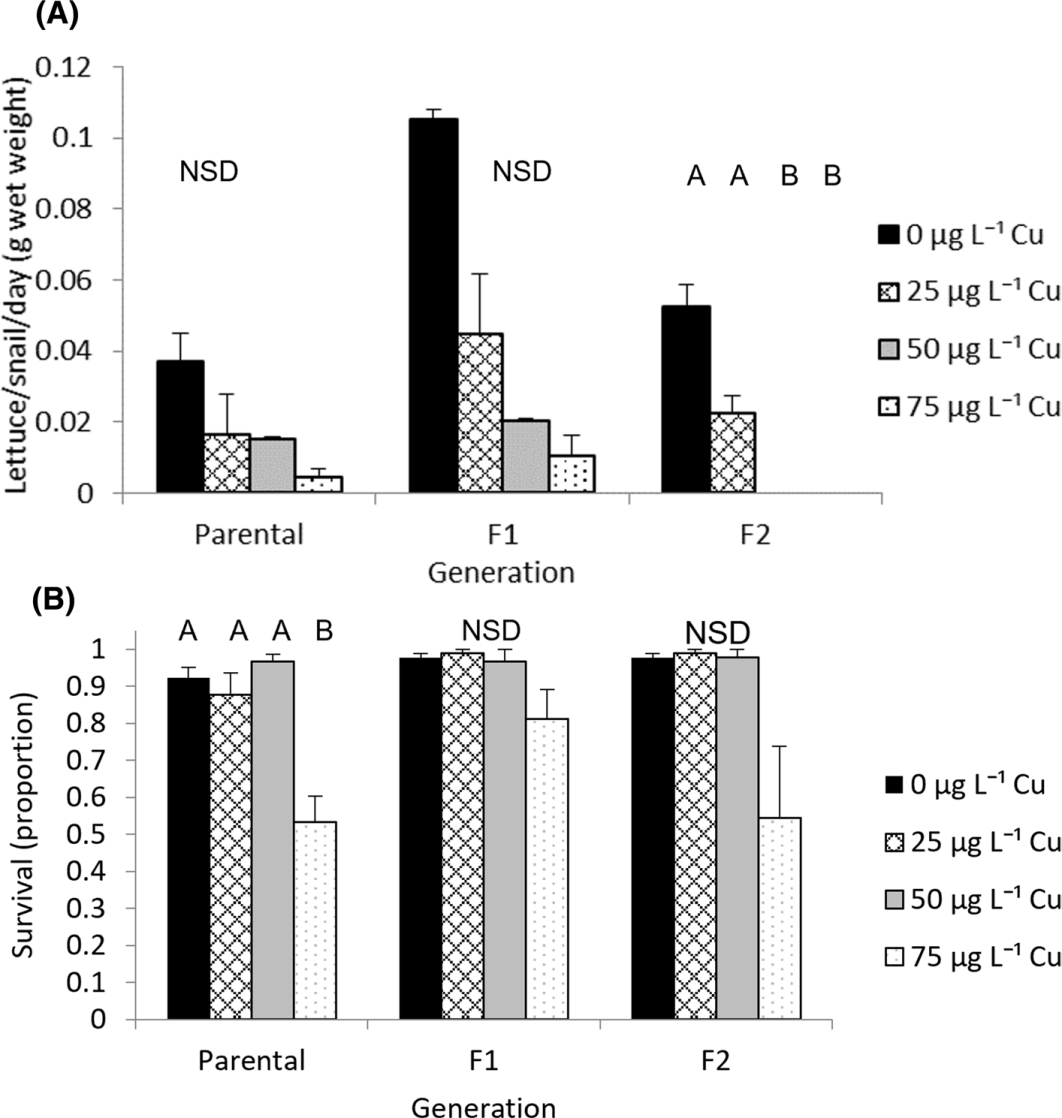
*Isidorella newcombi* exposed to Cu for 3 days **A** lettuce consumed, **B** proportion of exposed adults surviving. Letters on each graph indicate homogenous subsets; NSD = no significant difference (*P* > 0.05). Data are means and standard errors (*n* = 3 replicates of 30 snails per treatment)

In the parental and F_1_ generations, there was no significant difference between food consumption between treatments (parental *χ*^*2*^ = 6.44, *d.f.* = 3, *p* = 0.09; F_1_ *χ*^*2*^ = 7.51, *d.f.* = 3, *p* = 0.06). In the F_2_ generation, there was a significant difference in the food consumed by *I. newcombi* exposed to different Cu concentrations (*χ*^*2*^ = 10.65, *d.f.* = 3, *p* = 0.01). There was a strong negative correlation between Cu concentration and the amount of food consumed in the parental (*r*_*s*_ =  − 0.67, *n* = 12, *p* < 0.05), F_1_ (*r*_*s*_ =  − 0.80, *n* = 12, *p* < 0.01) and F_2_ (*r*_*s*_ =  − 0.93, *n* = 12, *p* < 0.001) generations (Fig. 2A).

### Adult Survival

#### Comparison Between Treatments Within Each Generation

There was a significant difference in the survival of *I. newcombi* exposed to Cu for 3 days in the parental generation (parental, *F* = 16.46, *d.f.* = 3, 8, *p* < 0.001). In the parental generation, the 75 µg L^−1^ treatment had significantly lower survival than all other treatments (*p* < 0.05). In the F_1_ and F_2_ generations, there was no significant differences in survival between the treatments (F_1_, *χ*^*2*^ = 4.91, *d.f.* = 3, *p* = 0.18; F_2_, *χ*^*2*^ = 7.03, *d.f.* = 3, *p* = 0.07). Despite the differences not being statistically significant, there was a trend of reduced survival in the 75 µg L^−1^ treatment compared to other treatments (Fig. 2B).

#### Comparison Between Generations Within Each Treatment

There were no significant differences in adult survival between the generations for the 0, 25 and 50 µg L^−1^ Cu treatments, but there was a significant difference in adult survival between generations in the 75 µg L^−1^ Cu treatment. In the 75 µg L^−1^ Cu treatment, the survival in the parental generation was significantly lower than that in the F_3_ generation (*F*_3, 8_ = 5.17, *p* = 0.03).

### Fecundity

#### Comparison Between Treatments Within Each Generation

**Fig. 3:**
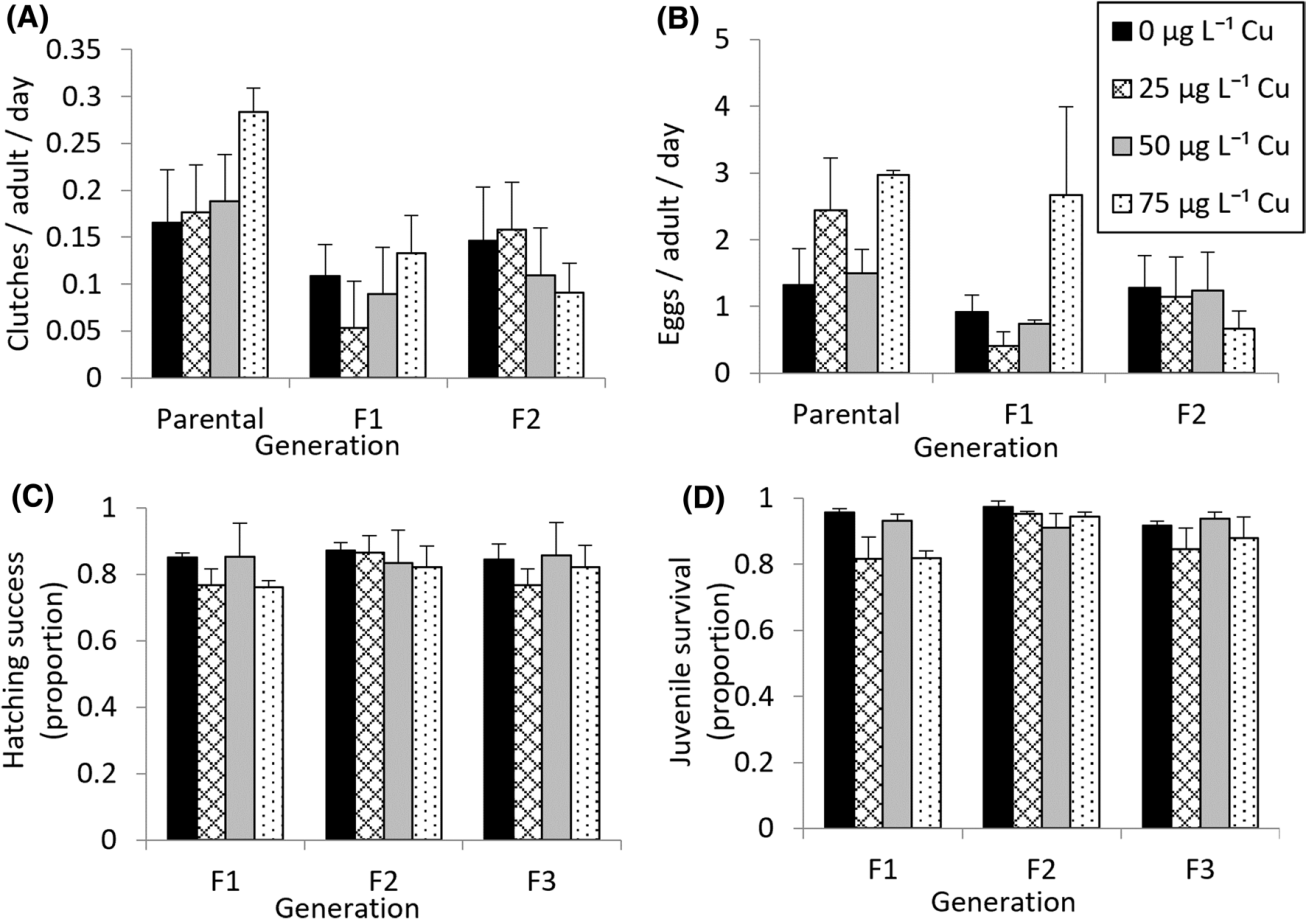
Exposure of *Isidorella newcombi* to 0 to 75 µg L^−1^ Cu for 72 h in the parental, F_1_ and F_2_ generations **A** Number of clutches **B** eggs laid, **C** Hatching success, **D** Juvenile survival by on days 15 to 26 days after exposure. Data are means and standard error (*n* = 3 replicates of 30 snails per treatment)

There were no significant differences in the number of clutches or the number of eggs laid by *I. newcombi* during days 15 to 26 after exposure to 0–75 µg L^−1^ Cu in any of the generations. There was a lot of variability in the clutches and number of eggs laid per adult between the treatments in the parental, F_1_ and F_2_ generations (Fig. 3A and B). There was also no regular concentration-dependent trend of increasing or decreasing clutch egg production per adult in the parental, F_1_ and F_2_ generations.

#### Comparison Between Generations Within Each Treatment

There were no significant differences in the number of clutches laid between generations for the 0, 25 and 50 µg L^−1^ Cu treatments, but there was a significant difference in the number of clutches laid between generations in the 75 µg L^−1^ Cu treatment (*F*_3, 8_ = 5.29, *p* = 0.03). In the 75 µg L^−1^ Cu treatment, significantly, more eggs were laid in the parental generation than in the F_2_ generation. In the 75 µg L^−1^ Cu treatment, there was a trend of a reduced number of clutches being laid after the parental generation, even though this was not statistically significant in the F_1_ and F_3_ generations (*α* = 0.05). Thus, in this treatment while not all generations laid significantly fewer clutches of eggs then the parental generation, there was a general trend of a reduction in clutch production in all subsequent generations (Fig. 3A). Despite the differences in the number of clutches, there were no significant differences in the number of eggs laid between generations in any of the treatments (Fig. 3B).

### Hatching Success

There were no significant differences in the hatching success of *I. newcombi* juveniles between treatments in any of the F_1_, F_2_ or F_3_ generations eggs laid by parents exposed to 0–75 µg L^−1^ Cu (Fig. 3C). There were no significant differences in the hatching success of juveniles from eggs laid by *I. newcombi* between generations for any of the treatments (Fig. 3C). There were no significant differences in the number of days to first hatching between treatments in any of the generations (SI2).

### Juvenile Survival

#### Comparison Between Treatments Within Each Generation

In the *F*_1_ generation, there was significant difference (*p* = 0.049) in the juvenile survival of *I. newcombi* (*x*^*2*^ = 7.82, *p* = 0.049, Fig. 3D), however, post hoc analysis indicated that there were no significant differences between any of the treatment pairs (*α* = 0.05). Given the significant result of the Kruskal–Wallis test, it is worth noting that if *α* was set at 0.1 for the post hoc analysis, the 75 µg L^−1^ treatment would have had significantly lower juvenile survival than the 0 and 50 µg L^−1^ treatments. There were no significant differences between treatments in the juvenile survival rate for the F_2_ and F_3_ generations.

#### Comparisons Between Generations Within Each Treatment

There were no significant differences in the juvenile survival of *I. newcombi* between generations within specific treatments (Fig. 3D).

## F_3_ Generation

### Bioaccumulation of Copper

**Fig. 4:**
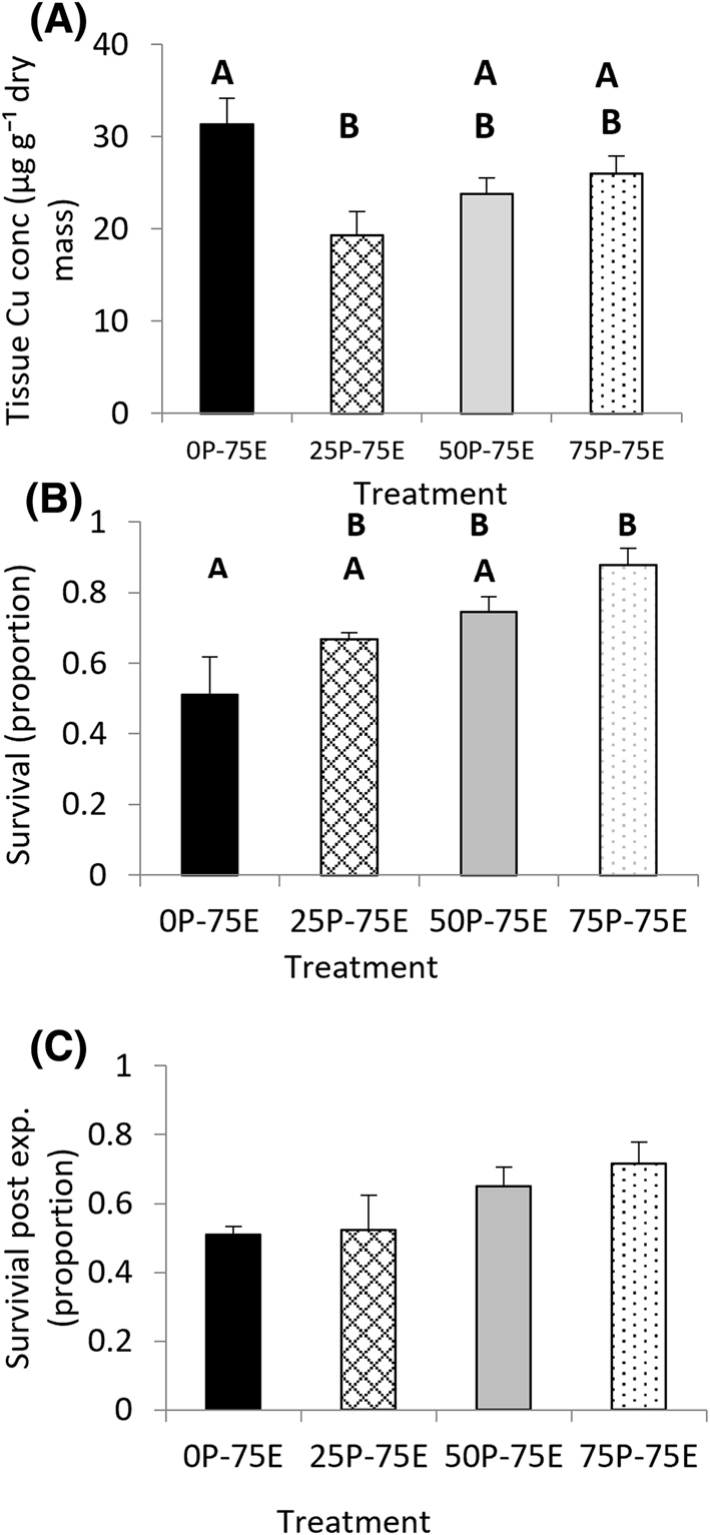
*Isidorella newcombi* with differing pre-exposure concentration histories **A** tissue Cu concentrations, **B** surviving, and **C** surviving for two weeks when exposed to 75 µg L^−1^ Cu for 72 h in the F_3_ generation. The labels on the *x*-axis indicate pre-exposure history and experimental exposure concentrations (P = pre-exposure, indicating the Cu concentration that the snails were exposed to in the parental, F_1_ and F_2_ generations, E = the Cu concentration that the snails were exposed to in the F_3_ generation. Letters indicate homogenous subsets within each parameter. Data are means and standard errors (*n* = 3 replicates of 30 snails per treatment)

When all treatments were exposed to 75 µg L^−1^ Cu for 72 h in the F_3_ generation, there was a significant difference in whole tissue Cu concentrations between the treatments (*F* = 10.83, *d.f.* = 3, 26, *p* < 0.01, Fig. 4A). The only treatments that were significantly different to each other in pairwise comparisons were 0P-75E and 25P-75E with the treatment with no pre-exposure history to elevated Cu concentrations accumulating higher concentrations of Cu. While they were not significantly different to each other, in the treatments that had pre-exposure to Cu, there was a trend of increased tissue Cu concentration with increasing Cu pre-exposure concentration histories.

### Survival (3 days Exposure)

In the F_3_ generation when all snails were exposed to 75 µg L^−1^ Cu, there was a significant difference in survival between the treatments based on their pre-exposure history (*χ*^*2*^ = 9.43, *d.f*. = 3, *p* = 0.02). The 0P-75E had significantly lower survival than the 75P-75E. There was a trend of increased survival with increased pre-exposure concentration history (Fig. 4B). A Spearman’s correlation confirmed that there was a strong correlation between F_3_ survival rates and pre-exposure concentration histories (*r*_s_ = 0.93, *n* = 12, *p* < 0.001).

### Survival: 14 Days Post-exposure

There was no difference in the survival rates of the snails in the 14 day after exposure to 75 µg L^−1^ Cu for 72 h in the F_3_ generation based on pre-exposure history (*F* = 2.25, *d.f.* = 3,8, *p* = 0.16). Despite no significant difference being detected between treatments, there is a trend of increasing survival in the treatments with a higher pre-exposure history (Fig. 4C). A Spearman’s correlation confirmed that there was a strong correlation between F_3_ post-exposure survival rates and pre-exposure concentrations (*r*_s_ = 0.73, *n* = 12, *p* < 0.01).

### Food Consumption

**Fig. 5:**
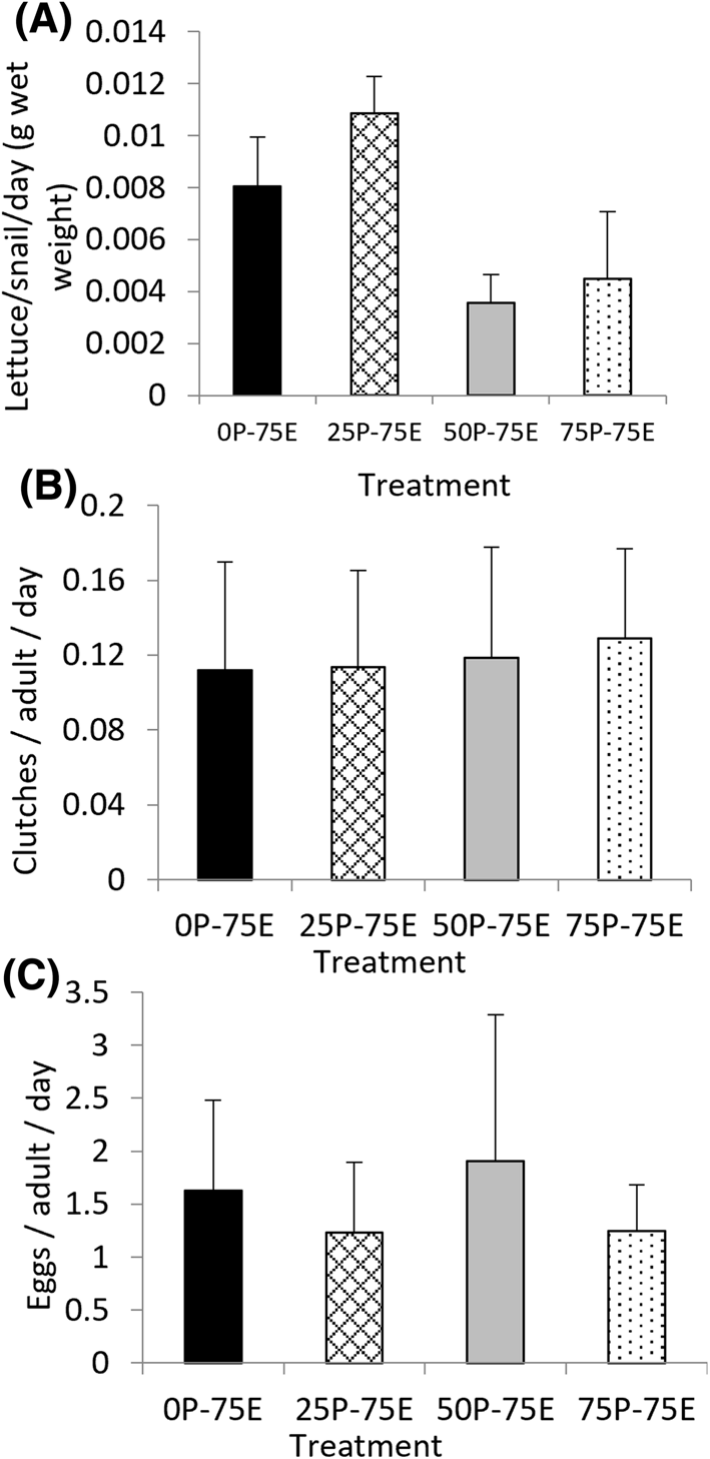
*Isidorella newcombi* exposed to 75 µg L^−1^ Cu for 72 h in the F_3_ generation **A** Lettuce consumed, **B** number of clutches, and **C** eggs laid. The labels on the x-axis indicate pre-exposure history and experimental exposure concentrations (P = pre-exposure, indicating the Cu concentration that the snails were exposed to in the parental, F_1_ and F_2_ generations, E = the Cu concentration that the snails were exposed to in the F_3_ generation). Data are means and standard errors (*n* = 3 replicates of 30 snails per treatment)

There was no significant difference between the food consumption of *I. newcombi* exposed to different Cu concentrations in the F_3_ generation (*χ*^*2*^ = 7.31, *d.f.* = 3, *p* = 0.06) (Fig. 5A).

### Fecundity

In the F_3_ generation, there were no significant differences in fecundity between treatments as assessed by the number of clutches (*χ*^*2*^ = 0.23, *d.f.* = 3, *p* = 0.97) or eggs (*χ*^*2*^ = 0.03, *d.f.* = 3, *p* = 0.99 (Fig. 5B and C); as there were insufficient snails to run a control (0P-0E) in the F_3_ generation, the number of clutches and number of eggs per snail per day from each treatment in the F_3_ generation were compared to the controls from each of the parental, F_1_ and F_2_ generations as a means of comparison against normal reproductive output. There were no significant differences between the number of clutches or eggs laid per adult per day in any of the treatments in the F_3_ generation and the control snails from the parental, F_1_ and F_2_ generations (clutches, *χ*^*2*^ = 1.0043, *d.f.* = 6, *p* = 0.99; eggs *χ*^*2*^ = 0.329, *d.f.* = 6, *p* = 0.99).

### Hatching Success

**Fig. 6:**
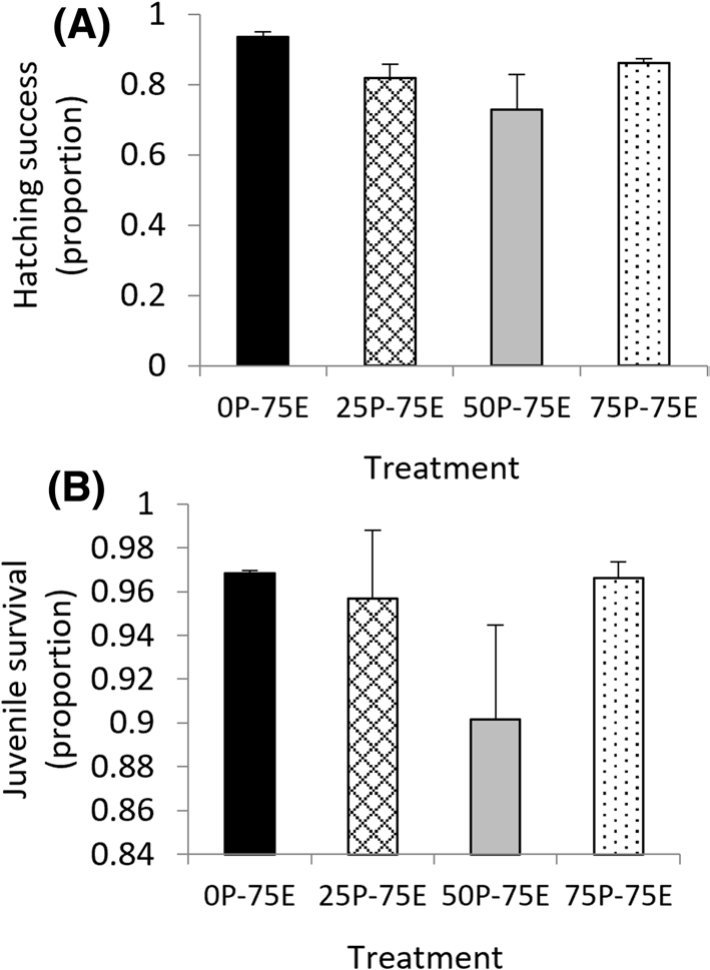
*Isidorella newcombi* 15 to 26 days after being exposed to 75 µg L^−1^ Cu for 72 h in the F_3_ generation **A** hatching success and **B** juvenile survival. The labels on the x-axis indicate pre-exposure and exposure concentrations (P = pre-exposure, indicating the Cu concentration that the snails were exposed to in the parental, F_1_ and F_2_ generations, E = the Cu concentration that the snails were exposed to in the F_3_ generation). Data are means and standard errors (*n* = 3 replicates of 30 snails per treatment)

In the F_4_ generation, there were no significant differences in juvenile hatching success between treatments (*χ*^*2*^ = 5.47, *d.f.* = 3, *p* = 0.14) (Fig. 6A); as no F_4_ control data were available due to insufficient snails, the F_4_ generation was compared against the controls for each of the F_1_, F_2_ and F_3_ generation. There was no significant difference between the hatching success of juveniles in any of the treatments in the F_4_ generation and the controls from any of the F_1_, F_2_ and F_3_ generations (*χ*^*2*^ = 7.08, *d.f.* = 6, *p* = 0.31).

### Juvenile Survival

In the F_4_ generation, there were no significant differences in juvenile survival between treatments (*χ*^*2*^ = 2.50, *d.f.* = 3, *p* = 0.48) (Fig. 6B). As no F_4_ control data were available due to insufficient snails, the F4 juvenile survival was compared against the controls for the F_1_, F_2_ and F_3_ generation. There was no significant difference between the proportion of juvenile survival in any of the treatments in the F_4_ generation and the controls from any of the F_1_, F_2_ and F_3_ generations (*χ*^*2*^ = 6.90, *d.f.* = 6, *p* = 0.33).

### Total Antioxidant Capacity

**Fig. 7:**
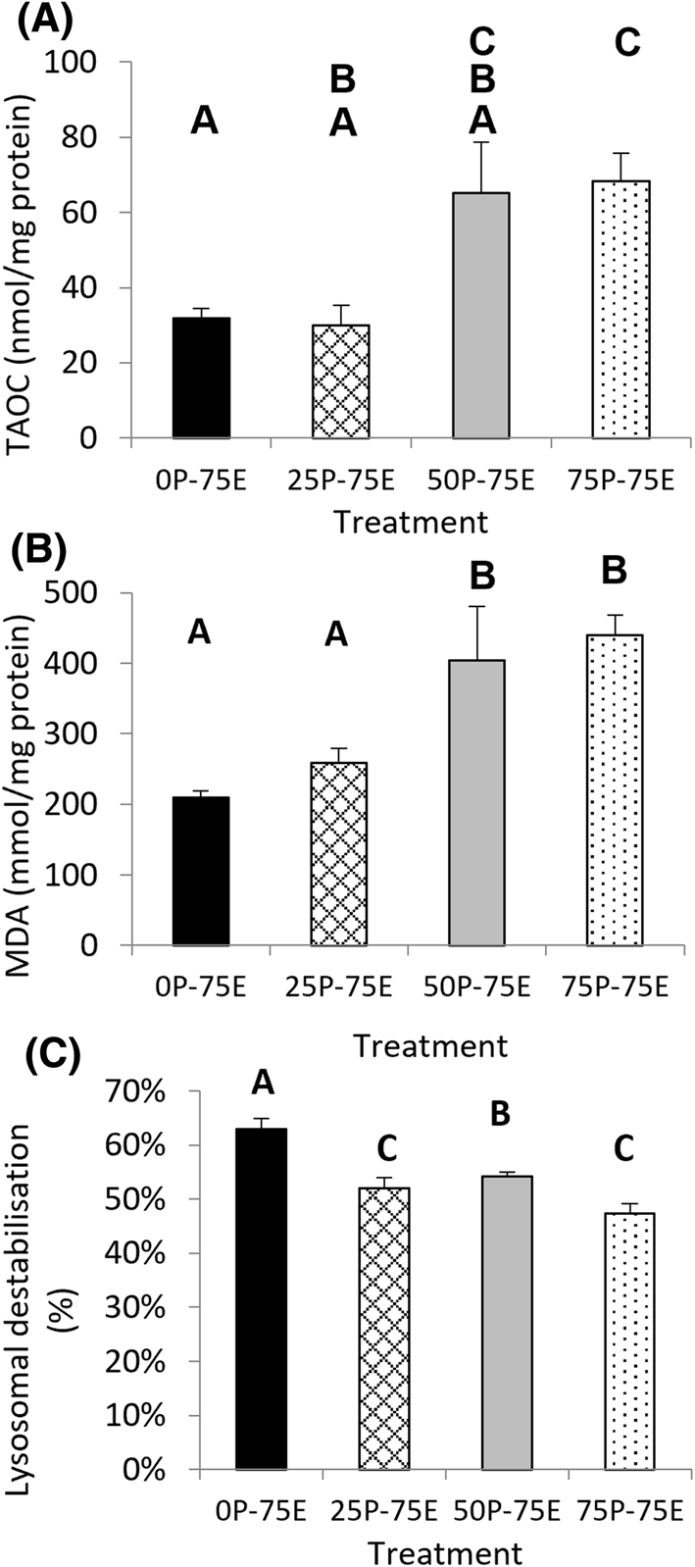
Biomarker responses, **A** total antioxidant capacity, **B** lipid peroxidation and **C** Lysosomal membrane destabilisation in *Isidorella newcombi* exposed to 75 µg L^−1^ Cu for 72 h in the F_3_ generation. The labels on the *x*-axis indicate pre-exposure history and experimental exposure concentrations (P = pre-exposure, indicating the cu concentration that the snails were exposed to in the parental, F_1_ and F_2_ generations, E = the Cu concentration that the snails were exposed to in the F_3_ generation. Letters on graphs indicate homogeneous subsets within each parameter. Data are means and standard errors (*n* = 9 individuals per treatment, consisting of 3 snails from each of the treatment replicates)

When all treatments were exposed to 75 µg L^−1^ Cu for 72 h in the F_3_ generation, there was a significant difference in TAOC between the treatments (*χ*^*2*^ = 10.83, *d.f.* = 3, *p* = 0.01). The treatments with lower pre-exposure history generally had lower TAOC than those that had been exposed to higher Cu concentrations in the parental, F_1_ and F_2_ generations (Fig. 7A).

### Lipid Peroxidation

When all treatments were exposed to 75 µg L^−1^ Cu for 72 h in the F_3_ generation, there was a significant difference in malondialdehyde (MDA) between the treatments (*χ*^*2*^ = 16.71, *d.f.* = 3, *p* < 0.001). As with the TAOC, the treatments with lower Cu pre-exposure history generally had a lower MDA levels than those that had been exposed to higher Cu concentrations in the parental, F_1_ and F_2_ generations (Fig. 7B).

### Lysosomal Membrane Destabilisation

In the F_3_ generation where all treatments were exposed to 75 µg L^−1^ Cu for 72 h, there was a significant difference in LD between the treatments (*F* = 14.7, *d.f.* = 3, 26, *p* < 0.001). The organisms that had no history of pre-exposure to Cu had the highest levels of LD, while the organisms that had been exposed to 75 µg L^−1^ in each generation of the study had the lowest levels of LD (Fig. 7C). The organisms that had been pre-exposed to 25 and 50 µg L^−1^ Cu did not follow the pre-exposure concentration-dependent decrease in LD in the F_3_ generation, however, the levels of LD in these treatments were very similar and there was no significant difference between these treatments.

### Relationships Between Biomarkers

There was a strong positive correlation between TAOC and malondialdehyde (MDA) in the F_3_ generation *I. newcombi* from all treatments. (*r* = 0.87, *n* = 27, *p* < 0.001). There was no relationship between TAOC and mean LD in *I. newcombi* from the individual replicates in the F_3_ generation (*r*_s_ = 250, *p* = 0.13). There was no relationship between mean MDA and mean LD in *I. newcombi* from the individual replicates in the F_3_ generation (*r*_s_ = 261, *p* = 0.07).

Relationships between cellular biomarkers and individual effects are given in Table 1. Adult survival was positively correlated to TAOC and MDA but negatively correlated to LD. Feeding rate was negatively correlated to TAOC. All other relationships between cellular biomarkers and individual level effects were not significant.

**Table 1:** Spearman’s correlation analyses between cellular biomarkers (total antioxidant capacity (TAOC), malondialdehyde (MDA) and lysosomal membrane destabilisation (LD) and individual effects (adult survival, mean number of clutches per snail, mean number of eggs per snail, proportion of eggs hatched (egg hatching) and juvenile survival) in the F_3_ generation of *Isidorella newcombi* sub-populations with differing Cu exposure histories, exposed to 75 µg L^−1^ Cu for 72 h

Variables		*p*	*rho*
TAOC	Adult survival	0.014	0.74
TAOC	Mean clutches	0.23	NA
TAOC	Mean eggs	0.086	NA
TAOC	Egg hatching	0.44	NA
TAOC	Juvenile survival	0.64	NA
MDA	Adult survival	0.015	0.74
MDA	Mean clutches	0.18	NA
MDA	Mean eggs	0.067	NA
MDA	Egg hatching	0.23	NA
MDA	Juvenile survival	0.61	NA
LD	Adult survival	0.005	-0.80
LD	Mean clutches	0.98	NA
LD	Mean eggs	0.57	NA
LD	Egg hatching	0.88	NA
LD	Juvenile survival	0.47	NA

Data used are for means of individual replicates

## Discussion

The experimental design for many multi-generational studies involves the exposure of organism to a constant concentration of a contaminant over the entire life cycle (e.g. Bal et al. 2017). While this may be realistic for some contaminants that are released continually, it does not reflect the way Cu is often released into the environment. Cu introduced to aquatic systems is known to adsorb to organic matter and sediments with bioavailable Cu quickly returning to background concentrations (Stevens et al. 2014). In many cases where Cu contamination occurs, bioavailable concentrations are likely to be elevated for a short time and then a return to background levels. The exposure conditions used in this study were designed to mimic a pulse event rather than exposure to a constantly elevated concentration. In this study, a 3 days pulse exposure was used when *I. newcombi* were approximately 60 days of age. In some studies that have exposed freshwater snails to Cu over at least a generation, exposures to constant Cu concentrations were used (Peña and Pocsidio 2007; Rogevich et al. 2008; Das and Khangarot 2011). In other studies with other taxa, pulse exposures have been used. Chen et al. (2012), for example, used an exposure model where fish were exposed to Cu at high, low and medium pulse exposure scenarios, to investigate changes in population growth rates and energy budgets, but the fish were exposed 12 × during their life. Diamond et al. (2005) used multiple pulsed exposures conducted over a 7 days period to investigate the effects of Cu, nitric acid, Cd and sodium chloride on early life stages of the fish *Pimephales promelas.* Hoang et al. (2007) used pulse exposures of Cu, Zn and Se to investigate mortality over a 21 days exposure period in *Daphnia magna.* While pulse exposures to Cu have been used in the past on other taxa, the current study is the only one to date that uses a pulse exposure to investigate the response of freshwater snails to Cu over multiple generations. It is also unique in using a single relatively short exposure within each generation over multiple generations to investigate effects.

### Parental to F2 Generations

#### Adult Survival

The 100 µg L^−1^ Cu treatment was discontinued after the parental generation exposure as a total of 6 individuals from the combined replicates survived the exposure period and 30 snails per treatment replicate were required for the next generation. This is similar to the lethal concentration calculated for the freshwater snail *Melanoides tuberculata,* where Cu was found to be the most toxic metal when the snail was exposed to a range of metals including Cu, Cd, Zn, Pb, Ni, Fe, Al and Mn, with a 96 h LC_50_ concentration of 140 µg L^−1^ Cu (Shuhaimi-Othman et al. 2012).

In the remainder of the treatments exposed to concentrations of up to 75 µg L^−1^ Cu, more than 50% of the snails survived in each treatment in each generation (Fig. 2B). The lethal concentrations in this study generally agreed with those from other studies. Reported values for LC_50_ Cu concentrations for freshwater snails range from 13 to 79 µg L^−1^ Cu (Watton and Hawkes 1984; Brix et al. 2011; Das and Khangarot 2011; Ng et al. 2011; Besser et al. 2016). There is some variability in the LC_50_ values reported in these studies which are likely to be associated with differing interspecific sensitivity, life stages of animals tested, water chemistry of media, temperature and exposure period. Watton and Hawkes (1984) calculated a 96 h LC_50_ value of 77 µg L^−1^ Cu for adult *Potamopyrgus jenkinsi.* While LC_50_ concentrations were not calculated in the current study, in each of the generations, more than 50 per cent of the snails exposed to 75 µg L^−1^ Cu survived and only 7 per cent of snails exposed to 100 µg L^−1^ Cu in the parental generation survived. This indicates that the LC_50_ would lie between 75 and 100 µg L^−1^ Cu, which is similar to the value calculated by Watton and Hawkes (1984) who also used adult snails and had a similar exposure period to that used in our study.

In the parental generation, *I. newcombi* exposed to 75 µg L^−1^ Cu had significantly lower survival than the other treatments. In the subsequent F_1_ and F_2_ generations, the 75 µg L^−1^ Cu treatment had lower survival than the other treatments, but not significantly so. A comparison of survival between the four generations exposed to 75 µg L^−1^ Cu showed that the F_3_ generation had a significantly higher survival rate than the parental generation. The increased survival at the same concentration in a later generation indicates that at this exposure concentration, the snails developed tolerance over the experimental period. A range of aquatic organisms have been shown to develop tolerance to Cu when exposed over multiple generations (Bossuyt and Janssen 2004; Kwok et al. 2009; Sun et al. 2014). All of these studies used longer exposure periods than in the current study, however, they are further evidence that evolutionary change can occur rapidly when populations are exposed to a contaminant that exerts strong selection pressure over multiple generations (Hoffmann and Hercus 2000). In the current study, the main purpose of the parental to F_2_ generations was to establish populations of *I. newcombi* that had different exposure histories to Cu so a comparison of the responses of the snails with different exposure histories could be undertaken in the F_3_ generation. The reduced survival in the 75 µg L^−1^ Cu treatments compared to the other treatments in these three generations was evidence of the high selection pressure being exerted by this treatment.

#### Food Consumption

During the parental, F_1_ and F_2_ generation exposures, only the F_2_ generation showed significant differences in the amount of food consumed. The use of the remaining wet mass of lettuce as the response variable limited the accuracy of the food consumption measurements, due to the effect of water on the lettuce weight, and some breakdown of the lettuce during the Cu exposures. Despite the lettuce mass being calculated against control lettuce left in media for 3 days rather than starting mass, this method still introduced variability into the data and combined with the small sample sizes, made the detection of statistically significant differences challenging. Despite this, the significant negative correlations between food consumption and exposure concentration in *I. newcombi* indicated that exposure to increased Cu concentrations affected snail feeding behaviour during the exposure period. There is prior evidence of exposure to high concentrations of Cu effecting feeding behaviour of freshwater snails. Feeding rates in the freshwater snail *Pomacea canaliculata* reduced when exposed to 67.5 µg L^−1^ Cu (Peña and Pocsidio 2007). *Lymnaea luteola* exposed to 56 µg L^−1^ Cu ceased feeding (Das and Khangarot 2011). While not measured, a general behavioural response of reduced movement at the high exposures was observed, with the snails in the 75 µg L^−1^ Cu treatment staying on the bottom of the aquariums, while the snails from the other treatments moved freely around the aquariums and fed. In the freshwater snail *Lymnaea luteola,* a reduction in locomotion has been reported in response to Cu exposure (Das and Khangarot 2011). The reduction in feeding may be associated with a broader behavioural response of reduced locomotion.

#### Fecundity

Fecundity was assessed in surviving snails two weeks after the exposure period. In various studies exposing freshwater snails to Cu, there has been a reduction in fecundity (Rogevich et al. 2008; Khangarot and Das 2010; Das and Khangarot 2011). These studies tested the direct effects of Cu on fecundity, whereas the current study tested the fecundity of surviving snails after exposure to assess trade-offs associated with developing Cu tolerance. There were no differences in fecundity when comparing the treatments within the generations. In the 75 µg L^−1^ Cu treatment when compared to the parental generation, there was a significantly reduced number of clutches laid per adult in the F_2_ generation and a trend of reduction in the number of clutches per adult in both the F_1_ and F_3_ generations. Despite the differences in the number of clutches in the 75 µg L^−1^ Cu treatment and the mean number of eggs per adult being lower in F_1_, F_2_ and F_3_ generations than the parental generation, there were no significant differences between these generations in the number of eggs laid per adult. So, while minor evidence of biological trade-offs in the form of reduced number of clutches laid was present in the snails from the 75 µg L^−1^ Cu treatment, this did not translate to a significant reduction in the number of eggs per snail and would have limited relevance at the population level. Significant biological trade-offs associated with resistance to metal have been reported in various species (e.g. Shirley and Sibly 1999; Mireji et al. 2010; Agra et al. 2011), however, in the current study, while there is some evidence of trade-offs occurring, they are unlikely to have an effect at the population level.

#### Egg Hatching Success and Juvenile Survival

There were no significant differences in either egg hatching success or juvenile survival, either between the treatments within a generation or between generations within any of the treatments. Direct effects of Cu exposure on freshwater gastropod eggs and juvenile survival have been reported (Khangarot and Das 2010). In the current study, there is no evidence of the exposure of the adults to Cu affecting the hatching success of eggs or juvenile survival.

### F_3_ Generation

In the F_3_ generation, all treatments were exposed to 75 µg L^−1^ Cu to allow a common baseline for the assessment of changes in tolerance and associated biological trade-offs resulting from previous exposure history. Analyses tested the differences between pre-exposure concentration rather than the exposure concentration itself. In the F_3_ generation discussion, the term pre-exposure concentration will be used to describe the Cu concentration that snails were exposed to in the parental to F_2_ generations.

#### Survival

In the F_3_ generation, there was a positive correlation between the pre-exposure concentration and survival during the three-day exposure period. This suggests that populations of *I. newcombi* developed some resistance that was related to the pre-exposure concentration of Cu that previous generations were exposed to. The only significant difference in survival was between 0P-75E and the 75P-75E snails.

#### 25 and 50 µg L^−1^ Cu Pre-exposure Treatments

The trend of increased survival in the F_3_ generation in the 25 and 50 µg L^−1^ Cu pre-exposure treatments as indicated by the correlation analysis is likely to be related to increased phenotypic plasticity as a result of prior Cu exposure rather than directional selection. In the parental, F_1_ and F_2_ generations of these treatments, there were high survival rates that were not significantly different to the controls. Additionally, there were no significant differences in the reproductive output between these treatments in the parental to F_2_ generations. Changes associated with directional selection would require tolerant individuals to make an increased contribution to the genetic make-up of subsequent generations. Given that there was no significant difference in mortality or change in reproductive output per adult, this seems unlikely. While changes associated with phenotypic plasticity are often short term, some of the epigenetic changes associated with phenotypic plasticity can be passed across generations (Head 2014). Genetic and epigenetic analysis would be the only way to show definitively that the changes in survival of these treatments were associated with intergenerational changes to phenotypic plasticity rather than directional selection, however, the weight of evidence strongly suggests phenotypic plasticity is the cause.

#### 75 µg L^−1^ Cu Pre-exposure Treatments

The increased survival of the snails from the 75P-75E treatment may be due to a number of factors. During the parental exposures, the 75 µg L^−1^ Cu exposed snails had significantly lower survival than the other treatments. In the F_1_ and F_2_ exposures, the trend of lower survival in the 75 µg L^−1^ Cu treatment continued, although there were no significant differences in these generations. Klerks et al. (2011) reported that when contamination affects survival or reproduction, natural selection will favour those individuals that are less sensitive to the contaminant. It is possible that the mortality of some of the snails in the 75 µg L^−1^ Cu treatment in the parental, F_1_ and F_2_ generations, was associated with genetic variability, with differential fitness resulting from variation in genotypes being the determining factor in their ability to survive the exposure. Under this scenario, directional selection could be expected in the 75 µg L^−1^ Cu pre-exposure treatment. In the 75 µg L^−1^ Cu pre-exposure treatment, the effect of intergenerational changes in phenotypic plasticity must also be considered. As the increases in survival in the F_3_ generation of the 25 and 50 µg L^−1^ Cu pre-exposure treatments appear to be associated with an increased phenotypic plasticity in these populations, it is possible that some or all of the increased survival in the 75 µg L^−1^ Cu pre-exposure treatment is also associated with increased phenotypic plasticity. It is likely that in the 75 µg L^−1^ Cu pre-exposure treatment, both mechanisms contributed to increased survival. It has previously been found that freshwater snails taken from differing field and laboratory populations with differing exposure histories to Cu had significantly different mortality when exposed to 65 µg L^−1^ Cu under controlled conditions (Côte et al. 2015). The current study further highlights the ability of freshwater gastropods with differing Cu pre-exposure histories to develop resistance and demonstrates that the development of resistance can occur in as little as 3 generations following 72 h Cu exposure within each generation.

In the F_3_ generation, the increased survival in the treatments that had pre-exposure histories to higher Cu concentrations continued in the 14-day recovery period directly after exposure when snails were maintained in uncontaminated water. This was indicated by a strong positive correlation between survival two weeks post-exposure and pre-exposure Cu concentration. This suggests that the snails that had a higher pre-exposure to Cu not only had a greater ability to survive the exposure period but had greater ability to recover after the exposure. In the freshwater snail *Pomacea paludosa* exposed to Cu in sediment, the highest mortality was associated with the treatment that bioaccumulated the highest Cu concentrations during the exposures and the increased mortality in this group continued after snails were removed from the exposure into uncontaminated water (Hoang et al. 2011). The findings from the current study indicate that resistance developed through generations of pre-exposure to Cu leads to increases in survival during this recovery period as well as during the actual exposure.

#### Food Consumption

In the F_3_ generation, there was no significant difference in lettuce consumption between the treatments. The small amount of lettuce consumed in all treatments in this generation was similar to consumption by the 75 µg L^−1^ Cu treatments in earlier generations. This suggests that despite the increased ability of *I. newcombi* with a high pre-exposure history to Cu to survive, their responses relating to important behavioural markers such as food consumption do not change with repeated exposure to this Cu concentration. This was further reinforced by an observed lack of locomotion in snails in all treatments during the F_3_ exposures, with snails spending most of the time at the bottom of the tanks. The reduction in feeding may be related to metabolic depression when the snails are exposed to high Cu concentrations. In another gastropod, the limpet *Siphonaria capensis,* a reduced metabolic rate measured by cardiac activity was reported in response to concentrations higher than 50 µg L^−1^ Cu (Marshall et al. 2004).

#### Bioaccumulation of Cu

While there were significant differences in the Cu bioaccumulation in the F_3_ generation, they did not follow a pre-exposure Cu concentration-dependent pattern. The 0P-75E and 25P-75E were the only treatments that were significantly different to each other. It was suggested by Morgan et al. (2007) that one mechanism by which tolerance to a chemical can be achieved is through reduced accumulation of that chemical. As the differences in the tissue Cu concentrations did not follow a pre-exposure concentration-dependent pattern or relate to differences seen in survival or LD, it is assumed that differences in bioaccumulation were not an important factor in differences in tolerances between populations.

#### Fecundity, Hatching Success and Juvenile Survival as Biological Trade-offs

It has been reported that there are biological trade-offs associated with increased tolerance to contaminants that manifest as reduced growth, reproductive output and survival compared to non-tolerant individuals in uncontaminated environments (Shirley and Sibly 1999; Xie and Klerks 2004). In the current study, biological trade-offs were assessed through fecundity (clutches and eggs per adult) of the surviving snails 15 to 26 days after Cu exposures. The juvenile hatching success and survival to six days were also assessed. Despite the differences in survival between the treatments in the F_3_ generation, there were no differences in either the number of clutches or eggs per adult, the hatching success or juvenile survival in this generation. The control treatment was exposed to the common concentration of 75 µg L^−1^ Cu in the F_3_ generation because there were insufficient snails for a full (0P-0E) control treatment. To compensate for the lack of a full control in this generation, the results for fecundity, hatching success and juvenile survival were also compared to the control results for these endpoints in the parental, F_1_ and F_2_ generations. No significant differences were found between the F_3_ results for these endpoints and the controls from earlier generations. This suggests that although the snails with high pre-exposure Cu concentration histories had developed tolerance to Cu as evidenced by the increased survival rates, there were no accompanying biological trade-offs. While trade-offs associated with resistance have been documented, there are also examples where this is not the case. In *L. stagnalis* from genetically different populations with varying pesticide exposure histories cultured in uncontaminated media, there was no significant reduction in egg production (Boue'tard et al. 2014). In malathion-resistant flour beetles, as opposed to a trade-off, it was reported that the resistant individuals had increased reproductive capacity (Arnaud et al. 2005). It has been noted that while biological trade-offs associated with resistance are often discussed in the literature, the presence of trade-offs and underlying mechanisms are not well understood and require further investigation (Medina et al. 2007).

#### Total Antioxidant Capacity

Total antioxidant capacity (TAOC) was measured in the F_3_ generation to investigate the effect of multiple generations of pre-exposure to Cu on the antioxidant response to Cu-induced stress. The TAOC of the F_3_ generation snails increased as the pre-exposure Cu concentration increased. This implies that the snails that had a higher Cu pre-exposure history had a greater ability to increase antioxidant function and neutralise reactive oxygen species (ROS). It is known that exposure to excess Cu increases ROS production in organisms (Livingstone 2001). Reactive oxygen species can lead to cellular damage in the form of lipid peroxidation, structural and functional changes to proteins, and damage to nucleic acids (Manduzio et al. 2005). The increase in TAOC in the snails with a pre-exposure history to high Cu concentrations would result in an increased ability to neutralise excess ROS generated by Cu interference in ROS homeostasis. On this basis, the increased antioxidant capacity of snails that had higher pre-exposure Cu concentration histories could play a role in the increased survival rate in the F_3_ generation.

#### Lipid Peroxidation

Lipid peroxidation (LP) occurs when organisms have high levels of ROS which interact with and breakdown membrane lipids (Livingstone 2001), and is, therefore, a measure of oxidative damage. The levels of LP in *I. newcombi* in the F_3_ generation increased as the pre-exposure Cu concentration history increased. In the snails with a multiple generation exposure history to high Cu concentrations, it was expected that LP would decrease, especially given that the level of total antioxidant capacity increased in the snails that had a history of high Cu concentration exposure. While our result was not expected, previous studies measuring LP in gastropods have also not fitted expected patterns. In previous work involving the marine gastropods *Nerita melanotragus* and *Bembicium nanum* subjected to a single 96 h Cu exposure, lipid peroxidation was lower in organisms exposed to high Cu concentrations than those exposed to low concentrations (R. Ubrihien, unpublished data).

In many studies investigating LP in gastropods, the response seems to be transitory. In studies that investigated exposures of less than 4 days, stress-induced changes in LP were present (Pannunzio and Storey 1998; Singh et al. 2008; Deschaseaux et al. 2010, 2011). Studies that investigated the LP response over a longer period have had mixed findings with some studies reporting there were no differences in LP (Reid and MacFarlane 2003; Sureda et al. 2009) and others finding that under long-term exposures LP continued to be elevated (Zhou et al. 2010; Cacciatore et al. 2015). Klobučar et al. (1997) found that changes to LP in *Planorbius corneus* exposed to pentachlorophenol were present but not dose-dependent after 2 days of exposure, were present and dose dependent after 8 days of exposure, and were not present at all after 13 days of exposure, illustrating the transient and inherent variability of the LP responses in gastropods. These studies have involved exposure to a wide range of stressors, and there are insufficient studies that have investigated the effect of Cu or even metals more generally on LP in gastropods to allow a useful comparison. The only consistent finding across studies is that gastropods exposed to oxidative stress-inducing chemicals over a short period of up to four days display a response. The reported responses are not always dose-dependent and the relative response between treatments can vary over time as demonstrated by Klobučar et al. (1997). In addition, the response can return to background levels over longer exposure periods (Klobučar et al. 1997; Pannunzio and Storey 1998; Sureda et al. 2009). While LP in *I. newcombi* in the current study did not follow the expected pre-exposure concentration-dependent pattern, given the reported transitory nature of LP responses in gastropods as well as the fact that reported responses have not always been dose-dependent the result is not without precedent. These findings, along with the reported results for LP in gastropods, highlight the need for time-course studies to better understand the regulation of the antioxidant system and associated oxidative damage when gastropods are exposed to oxidative stress-inducing chemicals.

#### Lysosomal Membrane Destabilisation

In the F_3_ generation, the 0P-75E snails that had no pre-exposure to elevated Cu concentrations had the highest LD and the 75P-75E snails which had the highest Cu pre-exposure history had the lowest. All treatments that had been pre-exposed to elevated concentrations of Cu also had significantly lower LD than 0P-75E. As LD has been recognised as an effective biomarker of contaminant-induced stress in molluscs (e.g. Moore et al. 1982, 2007; Ringwood et al. 2003; Broeg et al. 2005), this indicates that the snails from treatments that had been exposed to Cu in previous generations were under less stress than the snails that had no previous exposure to elevated Cu concentrations. As all treatments were exposed to a common Cu concentration in the F_3_ generation, the differences in LD must have occurred because of adaptions associated with the selection pressure exerted by exposures in the previous three generations. Changes in LD have been related to the accumulation of high concentrations of contaminants into the lysosome, the onset of programmed cell death and oxidative damage in the form of lipid peroxidation (Izagirre and Marigómez 2009; Johansson et al. 2010; Taylor and Maher 2010). While the snails from the 0P-75E treatment did accumulate the highest tissue Cu concentrations, it was only significantly higher than those from the 25P-75E treatment. Given that LD and tissue Cu concentrations followed a different pattern between treatments, it is unlikely the sequestration of Cu to the lysosomes was an important factor in the LD result. Previous studies in bivalve molluscs have linked LD to oxidative stress and the breakdown of lipid membranes through LP (e.g. Marasinghe Wadige et al. 2014; Taylor et al. 2016). In the current study, there is no correlation between LD and either TAOC or LP. On this basis, there is no clear evidence that the increased LD was as a direct result of oxidative stress. The other reported cause of increased LD is the initiation of programmed cell death mechanisms. Kiss (2010) reported the occurrence of apoptotic programmed cell death occurring in molluscs as a result of metal exposure. The underlying reason for the increase in LD is uncertain, however, there is clear evidence that the snails with a lower pre-exposure history to Cu are experiencing increased levels of general stress as indicated by increased LD.

#### Relationship Life History Traits and Cellular Biomarkers

In the F_3_ generation, adult survival was positively correlated with TAOC and LP and negatively correlated with LD. The differences in antioxidant capacity and LP in surviving organisms from each treatment may indicate reasons for the differential survival between treatments. The correlation between TAOC and survival is most likely due to increased TAOC allowing the neutralisation of ROS in the snails, with the associated reduction in oxidative stress leading to reduced mortality. In the bivalve mollusc *Mytilus galloprovinciallis* exposed to organophosphates, impaired glutathione redox status has been associated with an reduction in survival (Peña-Llopis et al. 2002).

The positive correlation between LP and survival is counter-intuitive. As LP is a measure of oxidative damage, it would be expected that increased LP would lead to increased mortality, and thus, our results were somewhat surprising. While the underlying mechanism for this response is outside the scope of this study, two potential mechanisms could be hypothesised. The first is associated with the differences in timing of the antioxidant response mechanisms, which may have led to differences in the onset of oxidative damage between the treatments that also had different TAOC. The second is that the snails with higher survival rates that had been exposed to Cu over multiple generations may have transferred resources to other biological functions that are more important to survival. While this study is not able to determine the exact reason, this response further demonstrates the need for integrated studies that investigate the mechanistic basis of biomarker responses. This is especially so for LP and TAOC that are widely used as biomarkers to interpret antioxidant responses and oxidative damage.

There was a negative correlation between adult survival and LD in the F_3_ generation. In the treatments that had been exposed to high Cu concentrations in the parental, F_1_ and F_2_ generations, there was increased survival and lower LD. As discussed, the reduced LD is likely to be related to increases in apoptotic programmed cell death. If organisms are experiencing sufficient stress-related damage that an increase in apoptosis is required, it is not surprising that there was also reduced survival in these treatments. LD is known to be a reliable biomarker of general stress (Broeg et al. 2005; Moore et al. 2007) and the correlation between increased LD and reduced survival in the treatments provides further evidence that this biomarker can predict changes at higher levels of biological organisation.

## Conclusions

In the parental to F_2_ generations, some general responses to short pulses of Cu were seen including reduced survival and reduced feeding in snails exposed to high Cu concentrations. These findings indicated that the snails from the high Cu exposure were under Cu-induced stress that would be likely to apply a selection pressure.

There was evidence that the selection pressures exerted on the snails in the parental to F_2_ generations led to adaptive changes, resulting in increased resistance to Cu. In the F_3_ generation, when all treatments were exposed to a common Cu concentration, there was an increase in survival that was correlated with the pre-exposure Cu concentrations of the treatments. The snails from treatments that had been pre-exposed to Cu also displayed a reduction in stress at a sub-lethal level as indicated through lower LD. While there were differences in the bioaccumulation of Cu in the F_3_ generation, they did not follow the same trend as increased survival, so it is unlikely that changes in bioaccumulation of Cu are leading to differences in tolerance. Changes in antioxidant response were also investigated as a possible mechanism for an increase in tolerance. While TAOC increased in the snails from the higher pre-exposure treatments, the oxidative damage marker LP followed the opposite trend. A review of oxidative stress responses in gastropods highlighted the need for further research in this area, as the responses often do not follow expected patterns and are different to closely related taxa such as bivalves. The mechanisms that led to the increase in Cu resistance in the treatments that had been previously exposed to high Cu concentrations are not clear.

This study also investigated the presence of biological trade-offs, in the form of reduced reproductive capacity, that are often reported to be associated with Cu-adapted populations. There was no evidence that there was any reduction in reproductive capacity in the treatments that had developed Cu resistance.

This study demonstrates that Cu resistance can develop over a short evolutionary time scale. While other studies have demonstrated the development of resistance over multiple generations exposed continuously to Cu, this study is unique in the use of a single short-term exposure to Cu in each generation. In aquatic environments, excess Cu introduced into the environment is often only bioavailable for a short period of time and this study demonstrates the potential for changes in resistance in populations exposed to Cu in this manner.

## Supplementary Information

Below is the link to the electronic supplementary material.

Supplementary file1 (DOCX 29 KB)
Detailed description of the total antioxidant capacity and TBARS biomarker methods.

Supplementary file2 (DOCX 554 KB)
Figure showing days to first hatching for Isidorella newcombi egg masses oviposited by I. newcombi exposed to copper concentrations over multiple generations. Adult snails were exposed for 3 days prior to maturity and egg masses were maintained in natural concentrations (number of egg masses ranges from 68-171).

## Data Availability

The datasets generated during the current study are available from the corresponding author on reasonable request.
