# Supplementary material for: Fitness of Isidorella newcombi Following Multi-generational Cu Exposures: Mortality, Cellular Biomarkers and Life History Responses
Source: Arch Environ Contam Toxicol. 2022 Apr 20;82(4):520–38. doi: 10.1007/s00244-022-00931-w (PMC9079030; doi:10.1007/s00244-022-00931-w)
Supplement: Supplementary file 1 — Supplementary file1 (DOCX 29 KB) Detailed description of the total antioxidant capacity and TBARS biomarker methods. [file 244_2022_931_MOESM1_ESM.docx]

The TAOC of tissue lysates was measured using a Cayman chemical assay (Cayman Chemicals, Michigan, USA, #709001). This assay is based on the ability of the antioxidants in the sample to inhibit the oxidation of 2, 2’-azino-di-[3-ethylbenzthiazoline sulphonate] (ABTS) to ABTS**^·^**^+^ by metmyoglobin. The samples were thawed at room temperature and 10 µL of each pipetted into a 96 well plate with 10 µL of metmyoglobin and 150 µL of ABTS^®^. Reactions were initiated with 40 µL of a 441 µM solution of hydrogen peroxide. The plate was shaken for 5 min at 25°C and absorbance was read at 750 nm on a BioRad Benchmark Plus® microplate spectrophotometer. The suppression of absorbance under reaction conditions in the sample is proportional to the concentration of combined antioxidants in the sample. The capacity of the antioxidant in the sample to prevent ABTS^®^ oxidation was compared to Trolox (Cayman Chemicals, Michigan, USA), a water-soluble tocopherol analogue. Sample antioxidant capacity is quantified as millimolar Trolox equivalents calculated from a 7-point Trolox standard curve.

Lipid peroxidation (LP) was determined by measuring the thiobarbituric reactive substances (TBARS) present in the tissue lysates. The Oxitek^®^ TBARS assay (Zeptometrix Corporation, Massachusetts, USA, #0801192) used is based on specificity of malondialdehyde (MDA), which is a by-product of lipid peroxidation, for TBARS. The MDA present in the sample forms a 1:2 adduct with the thiobarbituric acid in the reaction. Tissue lysates were thawed at room temperature and 100 µl of each was pipetted into pre-labelled glass test tubes. 100 µL of a 0.28M sodium dodecyl sulphate solution was added and the tubes were gently shaken to homogenize the solution. 2.5 ml of TBA / buffer reagent (0.5 g thiobarbituric acid dissolved in 50 ml acetic acid and 50 ml of sodium hydroxide) was added to each test tube. Samples were incubated in a water bath at 95°C for 60 min with glass marbles placed on top of the test tubes to prevent evaporation. After cooling in an ice bath for 10 minutes, samples were centrifuged at 2000 × g for 15 minutes at room temperature. Supernatants absorbances were read at 532 nm on a microplate spectrophotometer (BioRad, Benchmark Plus®). Absorbances were compared to those of an MDA standard prepared from 100 µM malondialdehyde bis (dimethyl acetal) made up in buffer. TBARS for individual samples were calculated as MDA µM equivalents from a 5 point MDA standard curve.

Protein in samples was measured to provide a baseline for the normalisation of TAOC and MDA. Protein was quantified using the Fluoroprofile® Protein Quantification Kit (#FP0010; Sigma Aldrich, USA), a fluorescent assay based on epicocconone. Fluorescence was read at 485 nm excitation and 620 nm emission wavelengths on a BioRad Benchmark Plus® microplate spectrophotometer. A bovine serum albumin (BSA) calibration curve was used to calculate protein concentrations.
