# Supplementary material for: Fitness of Isidorella newcombi Following Multi-generational Cu Exposures: Mortality, Cellular Biomarkers and Life History Responses
Source: Arch Environ Contam Toxicol. 2022 Apr 20;82(4):520–38. doi: 10.1007/s00244-022-00931-w (PMC9079030; doi:10.1007/s00244-022-00931-w)
Supplement: Supplementary file 2 — Supplementary file2 (DOCX 554 KB) Figure showing days to first hatching for Isidorella newcombi egg masses oviposited by I. newcombi exposed to copper concentrations over multiple generations. Adult snails were exposed for 3 days prior to maturity and egg masses were maintained in natural concentrations (number of egg masses ranges from 68-171). [file 244_2022_931_MOESM2_ESM.docx]

Supplementary Figure 1


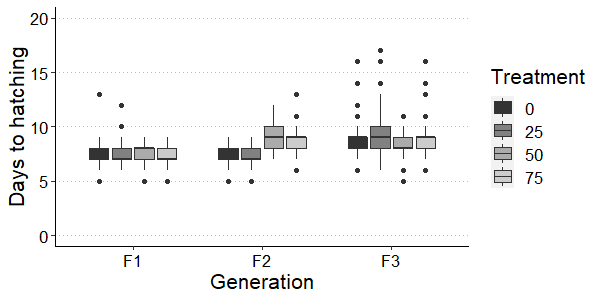


Figure 1. Days to first hatching for Isidorella newcombi egg masses oviposited by I. newcombi exposed to copper concentrations over multiple generations. Adult snail were exposed for 3 days prior to maturity and egg masses were maintained in natural concentrations (number of egg masses ranges from 68-171).
